# Drug Use Behaviors and the Risk of HIV Infection Among Drug Users in China Between 2014 and 2021: Cross-Sectional Study

**DOI:** 10.2196/56958

**Published:** 2024-09-10

**Authors:** Jiaqi Lv, Yangfan Jia, Chunhui Yan, Xingliang Zhang, Ke Xu, Junfang Xu

**Affiliations:** 1 The Second Affiliated Hospital, School of Public Health, Zhejiang University School of Medicine, Hangzhou, China; 2 Linping Campus, Second Affiliated Hospital, Zhejiang University School of Medicine, Hangzhou, China; 3 Department of AIDS/STDs Control and Prevention, Hangzhou Center for Disease Control and Prevention, Hangzhou, China

**Keywords:** drug user, drug-using behavior, HIV, behaviors, behavior, risky, sexual, drug, drugs, substance, STI, STD, sexual transmission, sexually transmitted, association, associations, correlation, correlations, sentinel, surveillance, sexually transmitted infection, sexually transmitted disease

## Abstract

**Background:**

Drug users are a high-risk group for HIV infection and are prominent HIV carriers. Given the emergence of new drugs, we explored current drug-using behaviors, HIV infections, and the correlation between drug-using behaviors and HIV infection risk among drug users from 2014 to 2021.

**Objective:**

We aimed to identify the prevalence of HIV infection risk among drug users and explore drug use behaviors based on the updated data, which could provide evidence for the precision of HIV prevention strategies among drug users.

**Methods:**

Data were collected from sentinel surveillance of drug users in rehabilitation centers and communities in Hangzhou (2014‐2021), including sociodemographic characteristics, HIV awareness, drug use, risky sexual behaviors, and HIV infection status. Multivariate logistic regression was used to identify the factors influencing HIV infection and risky sexual behaviors among drug users.

**Results:**

In total, 5623 drug users (male: n=4734, 84.19%; age: mean 38.38, SD 9.94 years) were included. New drugs dominated among the participants (n=3674, 65.34%). The main mode of drug use was noninjection (n=4756, 84.58%). Overall, for 27.45% (n=1544) of injected drugs in the last month before the investigation, the average daily injection frequency was 3.10 (SD 8.24). Meanwhile, 3.43% of participants shared needles. The incidence of sexual behaviors after drug use was 33.13% (n=1863), with 35.75% (n=666) of them using a condom in the last time. Overall, 116 participants tested positive for HIV antibodies (infection rate=2.06%). New drug users exhibited more postuse sexual behaviors than traditional drug users (odds ratio [OR] 7.771, 95% CI 6.126‐9.856; *P*<.001). HIV-aware drug users were more likely to engage in risky sexual behaviors (OR 1.624, 95% CI 1.152‐2.291; *P*=.006). New-type drug users were more likely to engage in unprotected sexual behavior (OR 1.457, 95% CI 1.055‐2.011; *P*=.02). Paradoxically, drug users with greater HIV awareness were more prone to engaging in unprotected sexual behavior (OR 5.820, 95% CI 4.650‐7.284; *P*<.001). Women engaged less in unprotected sex than men (OR 0.356, 95% CI 0.190‐0.665; *P*=.001). HIV rates were higher among injecting drug users (OR 2.692, 95% CI 0.995‐7.287; *P*=.04) and lower among drug users who used condoms during recent sex than those who did not (OR 0.202, 95% CI 0.076‐0.537; *P*=.001). Higher education levels were associated with higher HIV infection rates. However, there was no significant correlation between HIV cognition level and HIV infection.

**Conclusions:**

New drug types and noninjection were the main patterns in last 7 years. Using new types of drugs, rather than traditional drugs, was associated with an increased risk of HIV infection. Injection drug use was a risk factor for HIV infection. HIV awareness among drug users was high, but the incidence of risky sexual behaviors remained high. Therefore, it is important to promote the behavioral transformation of high-risk populations from cognition to attitude, and then to taking protective measures.

## Introduction

According to the Joint United Nations Programme on HIV/AIDS [1], 39 million people were living with HIV in 2022, with 1.3 million new HIV infections in that year. Drug use is an important underlying factor in the occurrence of risky sexual behaviors. The sharing of syringes among intravenous drug users is a direct cause of HIV infection and transmission. In 2019, HIV/AIDS was transmitted through the use of injection drugs in 10% of new cases globally [2]. Among the 15.6 million injection drug users (IDUs) worldwide, it was estimated that around 2.8 million (17.8%) were infected with HIV [3]. The HIV infection rate among drug users in various regions worldwide ranges from 1.1% to 35.6% [4]. Routes of drug use include the oral route, sniffing or intranasal usage, and injection [5]. Compared to other drug administration routes, substances have the strongest and fastest effects via intravenous injection [6].

Many early studies on IDUs have shown that most HIV infections in this population were caused by sharing needles, and sexual transmission can be ignored [7]. However, recent studies among never and former IDUs identified associations, suggesting that sexual transmission accounts for a substantial number of HIV seroconversions in these populations. In recent years, the use of new drugs (recreational drugs) such as new psychoactive substances has gradually surpassed the use of traditional drugs. These new drugs have strong euphoric effects and can reduce pain during anal sex among men who have sex with men and prolong the duration of sexual activity [8]. Most new types of drugs act on the central nervous system, stimulating the brain to release neurotransmitters such as norepinephrine, dopamine, and 5-hydroxytryptamine. Therefore, they are prone to producing extremely strong excitatory effects, enhancing the user’s sexual desire and pleasure, prolonging the duration of sexual behavior, increasing the frequency of sexual behavior, and predisposing individuals to unprotected sexual behavior with multiple partners as well as violent behavior, thus increasing the risk of HIV infection and transmission [9]. The relationship between the effects of drug use and sexual activity, such as libido increase, changes in body sensations, and decrease in social inhibitions, has been reported. The decrease in reflexes and planned choices associated with substance use may also lead to risky sexual intercourse [10,11]. Traceability studies on the transmission of HIV/AIDS have found that the paths of HIV infection and transmission among users of new types of drugs are mostly high-risk sexual behaviors after drug use [12]. With the change in usage from traditional to new types of drugs, sexual behaviors after drug use significantly affected the HIV infection rate by increasing the unsafe sexual practices [13-15].

HIV is mainly transmitted through blood, sexual contact, and mother-to-child transmission. For drug users, the main transmission routes are blood-borne and sexual transmission. Hence, we hypothesized 2 transmission routes for HIV infection risks among drug users. For intravenous drug users, blood-borne infection caused by unclean needles and needle sharing is the main mode of HIV transmission. For non-IDUs, sexual transmission caused by risky sexual behaviors is the main mode of HIV infection. Since new types of drugs are often consumed through noninjection methods and have strong hallucinogenic and neurotoxic effects, risky sexual behaviors after consuming new drugs are the main transmission routes of HIV infection. This study explored the prevalence of HIV infection risk among drug users and drug use behaviors based on the updated data, which could provide evidence for the precision of HIV prevention strategies among drug users.

## Methods

### Data Collection

Our study participants were drug users in rehabilitation centers and communities. The data were sourced from the monitoring data of drug use sentinel units (eg, the Public Security Bureau’s mandatory rehabilitation centers, regulatory facilities, methadone clinics, mandatory isolation rehabilitation centers, voluntary rehabilitation centers, and detention centers) in Hangzhou from 2014 to 2021. The drug users in sentinel monitoring were not exactly the same people annually. Sentinel monitoring refers to the selection of representative areas or high-risk groups (or both) based on the epidemiological characteristics of the monitored diseases to have a clearer understanding of the distribution of certain diseases in different regions and populations, as well as the corresponding influencing factors, and to carry out targeted, timed, and quantitative monitoring in accordance with the requirements of the National Sentinel Monitoring Plan. There are many national, provincial, and municipal HIV surveillance sentinels in Hangzhou, where 182 township hospitals and community health service centers were included.

Drug users refer to individuals who orally ingest, inhale, or inject drugs such as heroin, cocaine, opium, morphine, methamphetamine, cannabis, etc. The monitoring targets for drug users can be divided into 2 categories based on their source: drug users in custodial facilities and drug users in the community. The monitoring cycle is conducted annually. The sentinel surveillance period is from April to June. If the sample size met the surveillance requirements during the monitoring period, recruitment could be stopped. If the sample size was still insufficient at the end of the surveillance period, it could be extended for up to 1 month. The monitoring methods include on-site investigation and serological surveillance. The on-site investigation was conducted through a questionnaire survey using the “Health Survey Questionnaire.” Blood samples are collected from the target population, and the Hangzhou HIV Screening Center Laboratory was responsible for conducting HIV testing on the collected specimens and sending HIV-positive specimens for confirmatory testing.

The data included sociodemographic characteristics, HIV-related knowledge, drug use behavior, risky sexual behavior, and HIV infection status. A total of 5678 drug users were identified from 2014 to 2021, and 55 of them were excluded because they missed filling out some key variables (ie, risky sexual behaviors and drug abusing). Finally, 5623 drug users were included in the data analysis.

### Measurement

Drug-using behaviors were measured by drug type (ie, new or traditional drugs), patterns of drug use (ie, injection or noninjection), drug use status (ie, whether injection drug use has occurred in the last month and whether needles have been shared in the last month), and drug use frequency (average daily injection drug use frequency in the last month). Traditional drugs refer to heroin, opium, methamphetamine, morphine, marijuana, cocaine, and other narcotic drugs and psychotropic drugs regulated by the state that can make people addicted [16], mainly by injection and individual consumption. The new drugs mainly refer to artificial chemical synthesis of hallucinogens, stimulants, and other new psychoactive substances—also known as “club drugs,” mainly methamphetamine, ecstasy, K, hemp, happy water, etc, commonly found in parties, bars, nightclubs, and concerts [17]—that may lead to serious illness, injury, and even death.

HIV knowledge was measured using the 8-item HIV Knowledge Questionnaire, which has been widely applied in HIV-related surveys in China and has been proven to have good validity [18,19]. The knowledge awareness rate was determined in accordance with China’s HIV Prevention and Control Supervision and Evaluation Framework, and it is deemed to be known if it can correctly answer 6 or more of the 8 questions of the 8-item HIV Knowledge Questionnaire.

Risky sexual behaviors were measured by whether sexual activity has occurred after taking drugs and whether condoms were used during sexual activity after drug abusing.

Three HIV-related tests were conducted to determine the final HIV status. HIV antibody testing was carried out using enzyme-linked immunosorbent assay (ELISA) reagents. Western blotting was carried out to confirm an HIV-positive result.

### Statistical Analysis

Descriptive statistics were used to describe the basic characteristics, drug use behavior, and sexual behavior as well as HIV prevalence among drug users. Multivariate analyses were conducted to discuss the influencing factors (eg, drug use behaviors) of HIV infection risk measured by sexual behavior and unprotected sexual behavior after drug use, as well as HIV infection. As shown in the measurement, drug use behaviors measured by drug type, patterns of drug use, drug use status, and drug use frequency. In addition, the confounding factors considered in the multivariate analyses included gender, age, marital status, nationality, education level, and investigation year.

All data analysis was carried out using SAS (version 9.4; SAS Institute). Variables with *P*<.05 were considered statistically significant.

### Ethical Considerations

The study protocol and consent procedure were approved by Ethics Review Committee, School of Public Health, Zhejiang University (ZGL202306-9). The confidentiality of the study participants was properly protected during the investigation and data processing. Due to the use of deidentified data, the requirement for obtaining informed consent was waived. Access to the drug use sentinel units data is restricted to authorized individuals.

## Results

Among the 5623 participants, 84.19% (n=4734) of them were male and 15.81% (n=889) of them were female (Table 1). The average age of the drug users was 38.38 (SD 9.94) years. Most participants (n=2928, 52.07%) had an education level of up to junior school, followed by senior high school (n=1132, 20.13%), primary school (n=1056, 18.67%), college degree or above (n=299, 5.32%), and no formal education (n=214, 3.81%). Regarding marital status, 2081 (37.01%) participants were married, 2039 (36.26%) were unmarried, 1357 (24.14%) were divorced or widowed, and 146 (2.6%) were cohabiting participants. Among the participants, 105 (1.87%, 105/5623) were diagnosed with an HIV infection.

**Table 1.** Sociodemographic characteristics of the drug users (N=5623).

Items		Frequency
**Gender, n (%)**		
	Male	4734 (84.19)
	Female	889 (15.81)
**Age (years), mean (SD)**		38.38 (9.94)
**Marital status, n (%)**		
	Single	2039 (36.26)
	Married	2081 (37.01)
	Cohabiting	146 (2.60)
	Divorced or widowed	1357 (24.13)
**Nationality, n (%)**		
	Han Chinese	5314 (94.50)
	Minorities	309 (5.50)
**Educational level, n (%)**		
	Illiterate	214 (3.81)
	Primary school	1050 (18.67)
	Junior school	2928 (52.07)
	Senior high school	1132 (20.13)
	Junior college and above	299 (5.32)
**HIV status, n (%)**		
	Positive	105 (1.87)
	Negative	5518 (99.13)

In Table 2, the main type of drug use was that of new drugs (n=3674, 65.34%), while the proportion of participants using traditional drugs was 34.66%. In total, 867 (15.42%) participants have injected drugs. In the last month, 238 (27.45%) participants have injected drugs, with an average daily injection frequency of 3.10 (SD 8.24). Furthermore, 193 (3.43%) participants have shared needles with others. In the last month, 29 (15.03%) participants shared needles with others while injecting drugs. Among drug users who use traditional drugs, the majority of them (n=1310, 62.32%) adopt the pattern of injection for drug use. However, among users of new types of drugs, up to 97.87% (n=3446) adopt noninjection methods for drug use (Table 2).

**Table 2.** Drug use behaviors among drug users.

Patterns		Participants, %
**Drug type**		
	Traditional drugs	34.66
	New drugs	65.34
**Drug use patterns**		
	Injection	15.42
	Noninjection	84.58
**Ever injected in the last month**		
	Yes	4.23
	No	95.77
**Shared needles in the last month**		
	Yes	0.52
	No	99.48
**Have ever shared needles**		
	Yes	3.43
	No	96.57
**Frequency of sharing needles in the last month**		
	Never	99.48
	Sometimes	0.48
	Always	0.04
**Drug type and use patterns**		
	Traditional drugs (injection)	62.32
	Traditional drugs (noninjection)	37.68
	New types of drugs (injection)	2.13
	New types of drugs (noninjection)	97.87
**Drug type and shared needles**		
	Traditional drugs (shared needles)	90.90
	Traditional drugs (never shared needles)	9.10
	New types of drugs (shared needles)	0.10
	New types of drugs (never shared needles)	99.90

Among the 5623 drug users, 1863 (33.13%) had sexual intercourse after taking drugs in the last year, of whom 666 (35.75%) had used condoms during their last sexual activity after taking drugs, 2779 (49.42%) participants had sexual activity in the last month, of whom 1261 (45.38%) participants used condoms during their last sexual activity (Table 3).

**Table 3.** Incidence of sexual behaviors after drug and condom use among drug users.

Patterns		Participants, %
**Sexual behavior in the last year**		
	Yes	33.13
	No	66.87
**Sexual behavior in the last month**		
	Yes	49.42
	No	50.58
**Condom used in the last year**		
	Yes	35.75
	No	64.25
**Condom used in the last month**		
	Yes	45.38
	No	54.62

Table 4 shows that gender, age, marriage status, drug type, drug use patterns, investigation year, and HIV awareness significantly influenced sexual behaviors after drug use (*P*<.05). Specifically, compared to unmarried drug users, those who were married (odds ratio [OR] 0.662, 95% CI 0.506‐0.868) and those who were divorced or widowed (OR 1.437, 95% CI 1.039‐1.989) exhibited higher incidences of risky sexual behaviors. Users of new types of drugs reported higher incidences of risk behaviors than traditional drug users (OR 7.771, 95% CI 6.126‐9.856). Furthermore, drug users who are aware of HIV are more likely to engage in risky sexual behaviors than those who are unaware (OR 1.624, 95% CI 1.152‐2.291).

**Table 4.** Analysis of the influencing factors of HIV risky behaviors among drug users.

Drug types		Sexual behaviors after drug use^a^				Unprotected sexual behaviors after drug use^b^			
		B (SE)	Wald test	Adjusted OR^c^ (95% CI)	*P* value	B (SE)	Wald test	Adjusted OR (95% CI)	*P* value
New type of drugs (reference: traditional drugs)		2.050 (0.121)	285.73	7.771 (6.126‐9.856)	<.001	0.376 (0.164)	5.229	1.457 (1.055‐2.011)	.02
Noninjection drugs (reference: injection drugs)		0.174 (0.226)	0.595	1.190 (0.765‐1.853)	.44	−0.418 (0.350)	1.422	0.659 (0.332‐1.308)	.23
Shared needles (reference: not shared needles)		−1.060 (0.586)	3.274	0.347 (0.110‐1.092)	.07	−0.286 (0.563)	0.258	0.751 (0.249‐2.265)	.61
HIV awareness (reference: no HIV awareness)		0.485 (0.176)	7.639	1.624 (1.152‐2.291)	.006	1.761 (0.115)	236.506	5.820 (4.650‐7.284)	<.001
Female gender (reference: male)		−0.521 (0.153)	11.600	0.594 (0.440‐0.802)	.001	−1.034 (0.320)	10.463	0.356 (0.190‐0.665)	.001
Age		0.018 (0.008)	5.030	1.018 (1.002‐1.034)	.03	0.004 (0.014)	0.089	1.004 (0.978‐1.031)	.77
**Marital status (reference: single)**									
	Married	−0.412 (0.138)	8.938	0.662 (0.506‐0.868)	.003	−0.186 (0.243)	0.585	0.830 (0.516‐1.337)	.44
	Cohabiting	−1.240 (0.336)	13.617	0.289 (0.150‐0.559)	<.001	0.546 (0.840)	0.422	1.726 (0.333‐8.952)	.52
	Divorced or widowed	0.363 (0.166)	4.793	1.437 (1.039‐1.989)	.03	−0.386 (0.273)	2.008	0.680 (0.398‐1.159)	.16
Minorities (reference: Han Chinese)		−0.375 (0.211)	3.170	0.687 (0.455‐1.039)	.08	−0.708 (0.431)	2.698	0.493 (0.212‐1.147)	.10
**Educational level (reference: illiterate)**									
	Primary school	0.179 (0.318)	0.315	1.196 (0.641‐2.231)	.58	−0.953 (0.567)	2.828	0.386 (0.127‐1.171)	.09
	Junior school	−0.023 (0.303)	0.006	0.977 (0.539‐1.771)	.94	−0.083 (0.535)	0.024	0.921 (0.323‐2.627)	.88
	Senior high school	−0.447 (0.319)	1.968	0.640 (0.343‐1.194)	.16	0.382 (0.572)	0.446	1.465 (0.478‐4.496)	.50
	Junior college and above	−0.561 (0.359)	2.445	0.570 (0.282‐1.153)	.12	0.727 (0.618)	1.384	2.069 (0.616‐6.951)	.24
**Investigation year (reference: 2021)**									
	2016	1.824 (0.280)	42.350	6.196 (3.577‐10.732)	<.001	0.920 (0.154)	35.716	2.509 (1.856‐3.393)	<.001
	2017	3.681 (0.295)	155.545	39.691 (22.257‐70.784)	<.001	−1.661 (0.191)	75.578	0.190 (0.131‐0.276)	<.001
	2019	2.638 (0.265)	99.226	13.983 (8.322‐23.498)	<.001	−3.276 (0.361)	82.157	0.038 (0.019‐0.077)	<.001
	2020	2.177 (0.184)	140.228	8.817 (6.150‐12.641)	<.001	2.236 (0.285)	61.625	9.354 (5.353‐16.346)	<.001

a A total of 2986 individuals with missing values regarding post–drug use sexual behavior were excluded.

b A total of 3763 individuals who abstained from sexual activity for a year after drug use were excluded.

c OR: odds ratio.

Gender, drug type, level of HIV awareness, and investigation year significantly influenced unprotected sexual behavior (*P*<.05). Users of new types of drugs were more likely to engage in unprotected sexual behavior (OR 1.457, 95% CI 1.055‐2.011). Paradoxically, drug users with a higher level of HIV awareness were more prone to engaging in unprotected sexual behavior (OR 5.820, 95% CI 4.650‐7.284). The incidence of unprotected sexual behavior was significantly lower among women than among men (OR 0.356, 95% CI 0.190‐0.665; Table 5).

**Table 5.** Analysis of HIV infection among drug users.

Items		B (SE)	Wald test	Adjusted odds ratio (95% CI)	*P* value
New type of drug (reference: traditional drugs)		1.747 (0.410)	18.119	5.737 (2.567‐12.826)	<.001
Injection drugs (reference: noninjection drugs)		0.990 (0.508)	3.800	2.692 (0.995‐7.287)	.04
Sexual behavior after taking drugs in the last year: yes (reference: no)		−0.022 (0.298)	0.005	0.978 (0.545‐1.755)	.94
Used a condom during last sexual behavior in the last month: yes (reference: no)		−1.598 (0.498)	10.288	0.202 (0.076‐0.537)	.001
Used a condom during last sexual behavior in the last year: yes (reference: no)		0.200 (0.499)	0.160	1.221 (0.459‐3.244)	.69
Have HIV awareness (reference: no awareness)		0.133 (0.749)	0.031	1.142 (0.263‐4.959)	.86
Female gender (reference: male)		−0.445 (0.339)	1.717	0.641 (0.330‐1.247)	.19
Age		−0.019 (0.015)	1.677	0.981 (0.952‐1.010)	.20
**Marital status (reference: divorced or widowed)**					
	Single	0.546 (0.320)	2.910	1.727 (0.922‐3.234)	.09
	Married	−0.743 (0.379)	3.854	0.475 (0.226‐0.999)	.05
	Cohabiting	0.890 (0.680)	1.713	2.434 (0.642‐9.224)	.19
Han Chinese (reference: minorities)		0.20 (0.383)	0.272	1.221 (0.577‐2.585)	.60
**Educational level (reference: junior college and above)**					
	Illiterate	−18.687 (2934.883)	0.000	0.000 (0.000‐0.000)	>.99
	Primary school	−1.485 (0.314)	22.306	0.227 (0.122‐0.420)	<.001
	Junior school	−2.070 (0.281)	54.143	0.126 (0.073‐0.219)	<.001
	Senior high school	−1.457 (0.311)	22.000	0.233 (0.127‐0.428)	<.001
**Investigation year (reference: 2021)**					
	2014	−1.648 (0.565)	8.498	0.192 (0.064‐0.583)	.004
	2015	−0.180 (0.355)	0.259	0.835 (0.416‐1.673)	.61
	2016	−0.768 (0.516)	2.217	0.464 (0.169‐1.275)	.14
	2017	−0.852 (0.382)	4.972	0.427 (0.202‐0.902)	.03
	2019	0.210 (0.348)	0.364	1.233 (0.624‐2.439)	.55
	2020	−0.282 (0.341)	0.685	0.754 (0.386‐1.472)	.41

Compared to traditional drug users, new drug users also exhibited a higher susceptibility to HIV (OR 5.737, 95% CI 2.567‐12.826). Furthermore, injecting drug users exhibited a higher HIV infection rate than noninjecting drug users (OR 2.692, 95% CI 0.995‐7.287). Notably, drug users who used condoms during recent sexual intercourse within the last month had significantly lower HIV infection rates than those who did not use condoms (OR 0.202, 95% CI 0.076‐0.537). We found that a higher education level was associated with higher HIV infection rates. However, there was no significant correlation between HIV cognition level and HIV infection (Table 5).

## Discussion

We found that new types of drugs and noninjection drug use have become the mainstream. Compared to traditional drug users, new drug users were more prone to risky sexual behaviors. In addition, drug users with a higher level of HIV awareness were more likely to engage in risky behaviors. Multivariate analysis of HIV infection status showed that new drugs and injection drug use were risk factors. The level of HIV awareness was positively correlated with HIV infection.

The problem of abusing new types of drugs was becoming increasingly serious in China. At the end of 2010, there were over 1.5 million registered drug users nationwide, of which 430,000 (28%) reported using new drugs. Among the newly discovered drug users, approximately 120,000 (55%) participants used new types of drugs [16], and the majority were younger than 25 years. In 2015, China’s drug control report showed that the proportion of new drug users among the total drug user population stood at 57.1%, marking the first time that the consumption of new drugs surpassed that of heroin [20], which is consistent with our results.

Our study showed that there is a stronger correlation between new drug use and risky sexual behavior. Various studies reported that new drugs have significant stimulating and joyful effects. After consumption, there is often sustained brain hyperactivity. The use of new drugs before and during sexual activity may increase the likelihood of high-risk sexual behavior [21] (including increasing the number of sexual partners, prolonging sexual contact with partners, or reducing condom use [22]), thereby increasing the risk of HIV infection and transmission among users [23]. At the same time, due to the fact that new drugs are chemically synthesized, they are easier to produce, cheaper, and more convenient to take than plant extracts such as heroin [24], making them widely used. These new drugs are rapidly replacing heroin as the most common illegal drug in China. Our results show that the majority of drug users took the new drugs through the noninjection route, the risk of blood-borne HIV infection through injecting drug use is much lower than the risk of sexual transmission through risky sexual behavior. Efforts should be made to strengthen and prioritize the control of high-risk sexual behaviors among users of new drugs. Due to the unique characteristics of new type of drugs, public entertainment venues have become the main place for their popularity. Therefore, it is necessary to strengthen the management of public places such as bars and karaoke halls, carry out regular inspections and special crackdowns, and implement HIV prevention and management for people who often go to these places. Our results indicate that new drugs are a high-risk factor for HIV infection, which confirms the view that the use of new drugs can increase the risk of HIV infection and transmission among users [24]. This suggests that we should adopt more strict control over new drugs to reduce the incidence rate of HIV from the source.

In this study, noninjection became the main form of drug use. Since 2005, China has increased its efforts to crack down on traditional drugs such as heroin. The number of heroin users has shown a decreasing trend year by year, while new drugs have gradually replaced heroin. Due to the widespread use of new type of drugs users through nasal inhalation or swallowing, the number of injecting drug users has decreased, but currently, there is an increasing trend for drug users to inject new drugs in order to obtain stronger pleasure, which is consistent with reports from other countries [25].

Our results also indicate that injection drug use was a high risk factor for HIV infection, which is consistent with previous findings [26]. Because HIV is easily transmitted through the bloodstream, IDUs mainly spread HIV through sharing contaminated needles and syringes, as well as through their own risky sexual behavior. Participants in need and syringe exchange programs were associated with a substantively lower risk of HIV infection among intrauterine drug users in China [27]. Therefore, the management of injection needles is an effective measure to reduce the risk of AIDS transmission. Nowadays, the trend of drug users injecting new drugs for more intense pleasure has increased [28]. A study in San Diego, California, found that IDUs who think they are at higher risk of HIV infection were more likely to report sharing acceptable syringes and injection equipment [29]. Therefore, it is necessary to continue to promote the comprehensive implementation of community interventions such as drug maintenance treatment or clean needle supply and exchange, and to control the occurrence of risky behaviors among injecting drug users in order to reduce HIV infection caused by needle infection or needle sharing.

The proportion of drug users who had HIV knowledge in this study was relatively low (only 65%). In this study, HIV cognitive level was not a protective factor of risk of HIV infection. This may be attributed to subpopulations that are at a higher risk of acquiring HIV paying closer attention to AIDS-related knowledge, thus resulting in a higher level of AIDS awareness. For example, for groups of men who have sex with men as high-risk groups of AIDS, their HIV awareness rate was at a high level [30]. In addition, HIV awareness may not necessarily affect risk behavior practices, and participants with HIV knowledge may still engage in risky behaviors [31]. Behavioral intervention is more important for high-risk groups. Willingness to use HIV self-testing was significantly higher among drug users who had a high HIV risk perception than among those with low HIV risk perception [32]. People who had a higher awareness of pre-exposure prophylaxis had a higher willingness and actual usage rate to use it [33]. Therefore, social support for knowledge dissemination and behavior change should be more closely linked. According to the COM-B (Capability, Opportunity, Motivation-Behavior) model proposed by Micheie et al [34], in addition to the level of knowledge and physical ability, motivation and capability are also indispensable for behavior change. In addition to improving HIV awareness, we should provide high-risk groups with prevention and risk aversion measures (such as pre-exposure prophylaxis and HIV self-testing) to reduce the risk of HIV transmission among drug users.

Our findings should be interpreted with a number of limitations. First, our data source was limited to the Zhejiang region and has not been promoted nationwide. Second, as a socially sensitive topic, HIV/AIDS-related questions may be answered tenderly, leading to bias. Third, due to the national standardized uniform health behavior survey scale we adopted, there are still some variables (eg, occupation, duration of drug use, and related history) that may have an impact on the study variables, which need to be added in the future study. Finally, although our sample encompassed 7 years and the overall sample size was large, the sample size in a single year was small, and the incidence of risky sexual behaviors in different years was greatly different, which cannot effectively analyze the HIV infection risk situation in different years.

As the usage of new types of drugs continues to expand, it becomes imperative to enhance the management and oversight of individuals using these drugs, particularly given the increasing risky sexual behaviors associated with them, which serve as significant contributors to the spread of HIV. Injecting drug users continue to be a high-risk group for HIV infection; thus, promoting projects that enhance the availability of clean needles holds protective significance. Additionally, the risky sexual behaviors of injecting drug users are also deserving of attention. The study found that higher levels of HIV knowledge did not confer protective effects against HIV transmission and infection. High-risk groups for HIV, especially infected individuals, may pay more attention to related information, which may lead to a higher level of HIV knowledge. However, this level of knowledge has not been translated into safer sexual behaviors through motivation for self-protection or motivation to protect others. Additionally, increasing studies have found that information is a necessary but not sufficient condition for behavioral change. Individuals who have access to information and possess motivation to act still need to possess specific behavioral skills in order to translate information and motivation into actual preventive behaviors [35,36]. Interpersonal skills, especially the ability to maintain safe sexual practices even when faced with obstacles, are crucial factors in behavioral change among drug users. This underscores the importance of not limiting HIV prevention and control efforts solely to the cognitive level of knowledge dissemination, but rather emphasizing the more critical social processes of structural behavioral interventions.

## Abbreviations

COM-B: Capability, Opportunity, Motivation-Behavior
ELISA: enzyme-linked immunosorbent assay
IDU: injection drug user
OR: odds ratio
